# Supplementary material for: Are carbon emissions trading and green financial instruments synergistic? -Comprehensive quantitative research based on content analysis
Source: PLoS One. 2024 Mar 7;19(3):e0298601. doi: 10.1371/journal.pone.0298601 (PMC10919601; doi:10.1371/journal.pone.0298601)
Supplement: S1 Data — (ZIP) [file pone.0298601.s001.zip › Data/地级市Z_F工作报告.zip!/╕ú╜¿/┴·╤╥╩╨/└┤╫╘╣1⁄2╓┌║┼í╛┬φ┐╦╩2╛▌═°í┐_2019.docx]

各位代表：

　　现在，我代表市人民政府，向大会报告政府工作，请予审议，并请市政协委员提出意见。

　　一、2018年工作回顾

　　过去一年，在省委、省政府和市委的正确领导下，我市各级政府深入学习贯彻习近平新时代中国特色社会主义思想和党的十九大精神，按照中央、省经济工作会议部署，认真贯彻落实市委五届七次全会精神，坚持新发展理念，坚持高质量发展落实赶超，按照市委新时代新担当新作为“大督查大落实”要求，统筹推进稳增长、促改革、调结构、惠民生、防风险，经济社会发展取得新成效。

　　——全力稳增长，经济质量有新提升。完善落实工业21条、服务业20条、“一业一策”“一企一策”等政策措施，出台提升营商环境实施细则，全力推动经济发展提质增效。预计全市生产总值2386亿元、增长7.6%左右。第三产业增加值突破1000亿元，比重由“十二五”末的35.8%提升至42%。公共预算总收入297亿元、增长6.4%，地方一般公共预算收入151亿元、增长9%，地方税性收入占72.4%、同比提高5.3个百分点。预计城镇居民人均可支配收入35800元、增长8.4%，农村居民人均可支配收入17100元、增长9.3%。规模工业增加值增长9%，近七成企业产值实现两位数增长，企业利润总额增长35%以上。规模工业经济效益综合指数为420%，保持全省第一。高技术产业增加值增长18%，研发经费占GDP比重达1.62%，新增22家国家级、55家省级高新技术企业，入选国家创新型城市建设名单。上杭被评为全省县域经济实力十强县，长汀、连城、武平被评为全省县域经济发展十佳县。

　　——强力促改革，发展增添新活力。以利企便民促发展为导向，推出一批改革新举措，努力破解影响高质量发展的难题，疏通事关百姓切身利益的痛点堵点。

　　“放管服”改革力度加大。以e龙岩为抓手倒逼政务服务效能提升，手机端网办事项327项，“随手拍”受理诉求7.5万件、群众满意率达99%，在全国率先实现电子社保卡支付购药功能。e龙岩获得中国政府信息化管理创新奖、全省机关体制机制创新优秀案例一等奖，并在全省推广。由我市主导制定的全省“一趟不用跑”“最多跑一趟”政务服务规范标准发布实施。企业开办时间压缩至3个工作日以内。社会投资项目审批压缩至法定时限的40%以内。

　　重点领域改革取得突破。机构改革有序推进，部门设置、人员转隶工作基本完成。进一步加强和规范工程招投标、政府采购、财政投资评审管理。长汀水土保持综合治理模式被列为全国生态保护与修复工作典型。林权抵押贷款“惠林卡”模式在全国推广。完成农村土地确权登记颁证和农村集体产权制度改革清产核资。在全省率先开展农村实用人才职称评审。以担当作为精神解决中心城市1万多户房屋和土地登记历史遗留问题，得到自然资源部肯定并向全国推广。不动产登记中心被评为全国百佳不动产登记便民利民示范窗口。基本完成市属国有企业重组整合，组建市招商公司。代表福建省参与全国公立医院综合改革效果评价考核获得第一名。

　　——加快调结构，发展动能有新积蓄。集中优势资源，培育做大支柱产业，开展服务业“三比一看”竞赛活动，持续扩大有效投资，经济发展新动能加速成长。

　　“双培育”成效明显。有色金属、机械装备产业产值分别达到700亿元、430亿元，文旅康养产业总收入770亿元、游客超过4600万人次，建筑业产值突破1000亿元。50家重点培育的工业龙头企业产值增长19.8%，61家“一企一策”签约工业企业增长21.3%。龙净环保、龙马环卫入选“2018中国环境企业50强”。我市被授予“中国专用汽车名城”称号。

　　新产业新业态加快培育。“红古田”品牌影响力不断扩大，古田干部学院列入中组部第二批全国党员教育培训示范基地，红色教育培训超过19万人次。今日头条创作空间、内容质量中心落地龙岩，“跟着抖音游龙岩”反响热烈，与美团点评合作实施旅游生活化、智慧出行、食品安全监管等项目。成功创建国家新型工业化产业军民融合示范基地，军民融合项目新开工24个、新竣工13个，当年完成投资38亿元，民品参军26亿元。

　　投资后劲持续增强。固定资产投资增长13.5%，工业投资增长20%。“五个一批”、“重中之重”项目均超额完成年度投资任务。全年新签约项目285个、总投资915亿元。上杭太阳铜业连铸连轧、龙工挖掘机产能回归等一批项目竣工投产，长汀闪烁与激光晶体、万安溪引水工程等一批项目开工建设，南龙铁路、厦蓉高速扩容顺利通车，双龙铁路龙岩段可研获批，龙岩机场、漳平钢铁等重大项目前期取得突破性进展。

　　——突出惠民生，社会建设有新进步。持续加大投入，全市公共财政80%以上用于民生支出，28项为民办实事项目基本完成，就学、出行等问题得到较好缓解，群众获得感更加充实。

　　乡村振兴扎实推进。制定实施乡村振兴十条措施。以“质量提升年”为抓手，持续脱贫攻坚，创建激励性产业扶贫模式并在全省推广，建档立卡贫困人口基本脱贫，1个省级扶贫开发工作重点县、14个市级扶贫开发重点乡镇和264个贫困村可如期摘帽。扶持特色现代农业发展，七大优势产业全产业链产值突破700亿元。特色小镇和美丽乡村建设有序推进，在全省率先开展高标准农田“整乡整村”推进试点。加快“四好农村路”建设，完成农村路网改造355公里，配备352名乡村道路专管员。

　　城市面貌明显变化。持续实施中心城市畅通工程，新建及改造道路15公里，金鸡路三期、犀牛路二期等建成通车，龙岩大桥主塔桩基及承台已完成；打通8个交通拥堵节点，九一桥通行率提高2倍，西兴桥、罗桥通行率提高60%。全市新增公共停车位6400多个、人行天桥6座。持续实施中心城市园林绿化提升工程，落实城市绿化导则，新建成4处公园和41公里绿道，市民休闲健身增加许多新去处，龙津湖公园成为深受市民喜爱的城市网红新景观。注重文脉传承，恢复新罗第一泉、罗经泉，实施龙岩洞修复保护，组织开展龙津湖大家唱。

　　完成400多个无物业小区治理，整治搬迁韭菜园马路市场，解决了多年想解决而没有解决的难题。完善文明建设常态长效机制，推动文明行为促进条例立法和实施。上杭被列为全国新时代文明实践中心试点县。

　　社会事业不断提升。落实加强基础教育工作十条措施，组建专业化教育投资建设公司，启动应急工程项目管理机制，推进中心城市基础教育扩容项目建设。全市新改扩建中小学34所、新增学位1.8万个，新建公办幼儿园20所、新增学位7000多个。全年新增儿科床位150张、产科床位100张。基层医改深入推进，县域就诊率达到79%，“互联网+分级诊疗”列入科技部惠民项目。在省运会上获得64枚金牌。闽西现代汉剧《伯公灯》获省第七届艺术节一等奖。城镇登记失业率2.6%。城乡居民基础养老金提高至每人每月143元，惠及41万人。

　　与此同时，双拥、国防动员和后备力量建设进一步加强，妇女儿童、青少年、老年人、残疾人、社会福利、慈善和防灾减灾、对台事务、外事侨务、民族宗教、人防、地方志、档案、经济普查等各项事业取得新成绩。

　　——有效防风险，发展稳定有新保障。坚持精准施策，盯紧主要问题，强化综合治理，维护社会安定稳定，为高质量发展落实赶超提供坚实保障。

　　生态环保攻坚成果显著。扎实抓好中央、省环保督察反馈意见整改，集中整治环境突出问题。落实河（湖）长制，加强水污染防治，主要流域1—3类水质比例达95.5%，全部消除劣五类水质小流域，城市内河、县级以上集中式水源地、重点湖库水质达标率均为100%，小溪河水质持续稳定达标。开展大气污染企业专项整治行动，城市空气优良天数比例达98.9%，居全省前列。全市森林覆盖率达78.9%，保持全省第一。顺利通过创建国家现代林业科技示范园区评审。武平获评全国森林旅游示范县。环境治理工作获国务院大督查激励表扬。

　　金融运行稳健。发挥“政银司”协调机制作用，积极推动“增贷化险”，不良贷款率处于全省较低水平，金融机构贷款余额增速保持全省首位，信贷资产质量居全省前列。规范政府举债融资，市本级政府债务率连年下降。房地产市场保持平稳健康发展。

　　平安龙岩建设深入推进。扫黑除恶专项斗争取得阶段性成果，共侦办涉黑案件8起，打掉恶势力犯罪集团13个、恶势力犯罪团伙25个。涉麻制毒、电信诈骗、农村赌博和中心城区盗窃“三车”及电瓶专项整治成效明显，成功入围全国首批禁毒示范创建城市，长汀县摘掉全国涉麻制毒重点关注地区帽子。对安全隐患实行红、橙、黄、蓝分级管理、精准整治，安全生产形势总体稳定。食品安全全链条可追溯程度进一步提高。

　　打铁必须自身硬。我们坚持以习近平新时代中国特色社会主义思想为指导，坚持以党的政治建设为统领，树牢“四个意识”，坚定“四个自信”，坚决做到“两个维护”，全力推动中央、省和市委决策部署落地生根见效。坚持在市委统一领导下开展工作，按照新时代新担当新作为“大督查大落实”要求，强化“主责人、主办人”意识，提振干事创业精气神。开展机关作风整治，推动正向激励和逆向惩戒常态化，全面实行市直单位内部二级绩效考评，通过e龙岩让群众直接评价政务服务，提高政府工作效能。“七五”普法和宪法学习宣传深入开展。省委巡视反馈问题逐项整改落实。审计监督、效能督查持续深化。政府系统党风廉政建设和反腐败工作扎实推进。

　　各位代表，市政府及其各部门自觉接受市人大及其常委会的监督、市政协民主监督、社会监督，加强与人大、政协工作联系，主动向市人大及其常委会报告工作，重大事项及时和人大、政协沟通，认真执行人大决议决定，听取采纳人大代表建议和政协委员提案，由市政府领导牵头办理重点建议、重点提案，不断改进和推动政府工作。一年来，我们共办理人大代表建议345件、政协委员提案423件，办理质量进一步提升。按月抵冲住房公积金贷款、征迁安置小区不动产登记、扶持中小微企业发展等建议，已转化落实为具体政策措施，推动解决了一批群众关切的“民生大事”，更好推动高质量发展。

　　各位代表，过去一年取得的成绩，是坚持以习近平新时代中国特色社会主义思想为指引的结果，是省委、省政府和市委正确领导的结果，是全市人民凝心聚力、攻坚克难和各方面大力支持的结果。我代表龙岩市人民政府，向全市人民，向人大代表、政协委员，向各民主党派、各人民团体和各界人士，向中央和省直驻岩机构、驻岩部队和武警官兵，向关心支持龙岩发展的港澳台同胞、海外乡亲和国际友人，向为我们打下良好工作基础的老领导、老同志，表示诚挚感谢！

　　同时，我们更加清醒地看到存在的困难和问题，主要是：经济转型升级的动力仍然不足，重点产业规模不大、层次不高、龙头不强，大项目好项目较少，新的财源点不多，一些企业生产经营困难；群众最关切的操心事、烦心事尚未得到根本解决，基本公共服务和社会保障短板仍然较多，优质教育资源不够均衡，就医环境有待改善，城乡、区域发展不平衡不协调，乡村振兴基础较弱，平安建设、安全生产、生态环保等方面还存在重大薄弱环节；政府工作还有许多不足，服务能力和水平有待提高，一些政策措施没有落到实处，机关作风“六大方面”问题在政府系统中仍然存在。对此，我们决不能回避，必须勇于担当、砥砺攻坚，不断提升政府工作，全力以赴推动高质量发展落实赶超。

　　二、2019年工作安排

　　2019年是新中国成立70周年，是古田会议召开90周年和全军政治工作会议召开5周年，是决胜全面建成小康社会的关键之年，也是坚持高质量发展落实赶超的关键之年。习近平总书记高度重视、十分关心闽西发展，先后20次到闽西考察指导工作，从经济社会发展、人民群众生活、生态文明建设、全面从严治党等各方面亲自关心、指导、推动闽西苏区的建设与发展。习近平总书记强调“我们永远不要忘记老区、永远不要忘记老区人民”，并多次作出重要指示批示，高度肯定水土流失治理“长汀经验”和林改“武平经验”并要求在全国推广。习近平总书记对闽西发展寄予殷切期望，充分体现了对闽西人民的深厚感情，更为我们推进闽西各项事业发展提供了根本遵循。中央进一步加大力度支持原中央苏区振兴发展，省委作出坚持高质量发展落实赶超和进一步深化改革扩大开放的重大部署。这些为我市加快发展指明了方向，提供了新机遇，增添了新动力。

　　我们必须坚持以习近平新时代中国特色社会主义思想为指导，深入贯彻党的十九大精神，按照“五位一体”总体布局和“四个全面”战略布局，坚持稳中求进工作总基调，坚持新发展理念，坚持以供给侧结构性改革为主线，贯彻“巩固、增强、提升、畅通”方针，落实“六稳”工作要求，紧紧围绕高质量发展落实赶超，认真贯彻落实市委五届七次、八次全会精神，进一步深化改革扩大开放，持续打好三大攻坚战，扎实开展“产业发展项目建设年”活动，着力壮大产业，着力项目攻坚，着力民生民忧，着力城乡协同，着力改革创新，加快建设机制活、产业优、百姓富、生态美的新龙岩。

　　全年经济社会发展主要预期目标是：全市生产总值增长 8～8.5%；一般公共预算总收入增长5.5%，地方一般公共预算收入增长4.5%；固定资产投资增长10%左右；外贸出口增长3%，实际利用外资增长6%；社会消费品零售总额增长11%，居民消费价格总水平涨幅3%左右；城镇登记失业率控制在4.2%以内；城镇居民人均可支配收入增长9.3%，农村居民人均可支配收入增长9.3%；完成节能减排降碳任务。

　　为此，重点做好以下五个方面工作：

　　（一）着力壮大产业，夯实发展基础

　　产业是推动高质量发展落实赶超的基石。按照“五基地六产业七景区”发展布局，抓龙头、铸链条、建集群，全力培育支柱产业、龙头企业，打造龙岩产业升级版。

　　1. 加快融入“两区”建设发展。主动融入闽西南协同发展区，进一步放大协同效应，重点推进产业对接、交通互联、山海协作等15个方面重点任务和111个重点项目建设，突出抓好双龙铁路龙岩段、龙岩机场等重大项目。积极融入粤港澳大湾区建设，把粤港澳大湾区作为招商引资主阵地，发挥我市交通区位、商缘文缘、绿色生态等优势，开展常态化招商活动，重点在先进制造业、新兴产业、现代服务业等领域深化对接合作，引进一批高端优质项目。

　　2. 持续实施“双培育”行动计划。围绕培育有色金属、机械装备、文旅康养3个千亿级支柱产业和100家10亿元以上企业、10家百亿企业，精准实施“一业一策”“一企一策”，进一步集中优势资源，发展壮大一批支柱产业和龙头企业，推广上杭抓产业链的好经验，支持推动县（市、区）打造百亿产业集群。高质量推进有色金属产业，提高产业链水平，重点抓好紫金铜业铜冶炼40万吨技改、紫金山环境安全整体提升工程、卓尔稀土永磁钐钴合金二期等项目，打造金铜产业基地和稀土产业绿色发展基地，力争有色金属产业产值实现840亿元以上。高质量推进机械装备产业，提升中国专用汽车名城和国家应急产业示范基地品牌效应，重点发展高端专用车、工程机械和环保装备、应急装备，加快推进龙工锻件生产、龙净环保输送装备及智能制造、龙马环卫扩建、漳平钢铁、新龙马重组等项目建设，力争机械装备产业产值突破500亿元。高质量推进文旅康养产业，突出红色、生态、客家三大主题，建设提升“七景区”，重点抓好龙岩洞文化创意产业园、红炭山健康养老基地、中央苏区红色交通线、冠豸山创5A级旅游景区等项目；持续唱响“红古田”，以古田干部学院为龙头，做大做强教育培训、文化创意等红色产业链，建设“红古田厅”，开拓“研学旅行”，提升旅游消费质量，打造“红色文化之都”，力争文旅康养产业总收入突破1000亿元。

　　加快发展新材料新能源产业，重点打造稀土新材料、新能源材料、氟化工材料、硬质合金材料等四大产业链，突出抓好天甫半导体级电子材料等项目建设，力争产值突破150亿元。大力发展特色现代农业，建设省级现代农业产业园，打造“红古田”农产品区域公用品牌，培育特色农业+文化旅游、+休闲康养等新业态，促进农村一二三产业融合发展，力争优势产业全产业链产值760亿元以上。抓好粮食生产。推进产业数字化、数字产业化，实施“互联网返乡工程”，深化与今日头条、美团点评等互联网知名企业对接合作，培育电子商务、大数据、物联网、共享经济等数字产业，打造数字经济产业基地。

　　支持龙烟做大“古田”品牌，加快烟草片烟醇化库建设，推进烟草产业提质增效。持续做大做强建筑产业，推动装配式建筑发展，支持符合条件企业申报特级资质，培育一批产值30亿元以上龙头企业。提质发展建材产业，推进水泥企业智能化改造，积极发展新型建材，抓好永定红石材循环经济产业园、龙泰新能源等项目建设，力争实现产值320亿元以上。深入推进国家新型工业化产业军民融合示范基地建设，力争军民融合项目新开工20个以上、新竣工10个以上，实现民品参军25亿元以上。

　　推进“双百”培育工程，优化融资、市场、人才等服务保障，重点推动“老树发新枝”，新增10亿元以上企业8家、省级“专精特新”企业18家，百家成长型中小微工业企业产值增长35%以上。新培育规模以上服务业企业30家、限额以上商贸企业50家。

　　3. 大力支持民营企业发展。让民营企业充分感受到“自己人”的温暖，构建亲清新型政商关系。聚焦企业关切，对接企业需求，提高惠企政策精准度、获得感。主动帮助企业有效解决融资、用工、市场开拓等方面的实际困难，落实减税降费措施，降低民营企业用地用电用气成本，对诚信守法企业实行“无事不扰”，切实减轻企业负担。推动金融机构加大对民营企业信贷支持力度，落实好民营企业贷款“一二五”政策和小微企业“两增两控”目标，提高民营企业无还本续贷笔数及金额占比，拓宽银行抵（质）押范围，加大对民营企业“过桥”转贷支持。禁止对民间投资单独设置附加条件、歧视性条款和准入门槛，鼓励和支持社会资本以特许经营、参股控股等方式，参与养老、医疗、教育、文体等项目建设运营。开展质量提升行动，鼓励企业加强品牌建设。激发和弘扬企业家精神，更加爱护和支持企业，常态化组织企业家参加高层次培训，鼓励有条件的民营企业建立现代企业制度，聚焦实业做精主业，激发民营经济创新创造活力。

　　4．有效破解融资难题。推动银企融资精准对接，加强投融保联动，扩大中小企业信贷规模。探索对中小微企业信用建档，解决融资担保信用资信不足问题。支持优质企业股改上市、优化资产，重点推动龙岩高岭土、天泉药业、水发环境、侨龙专用车等后备企业上市。鼓励符合条件的工业企业通过公司债、私募债、短期融资券、中期票据等，开展债务融资。发挥政府性投资带动作用，进一步扩大社会投资。支持国有企业以基金运作、股权合作等方式，市场化运作优质产业项目。鼓励国有企业与社会资本探索组建投资经营联合体，共同推进基础设施、公共服务等项目建设。积极向上争取基础设施、专项债券、彩票公益金等项目资金支持。

　　（二）着力项目攻坚，增强发展后劲

　　项目是经济工作的生命线和主要抓手。必须强化要素保障，突出抓好“三个100”项目，培育更多增长点、拉动点和增收点。

　　1．推进项目滚动发展。落实省“五个一批”要求，加强动态管理，紧盯关键环节，推动一批重大项目生成、转化、落地。突出抓好100个增加值增长点、100个投资增长点、100个税收增长点项目，全力攻坚60个“重中之重”项目，确保上杭常青三元前驱体、双龙铁路龙岩段等项目开工建设，中石油（长汀）裂化催化剂、永杭高速等项目投产或建成，加快靖永高速、万安溪引水工程等项目建设，力争龙岩机场早日立项和开工建设，做深做实龙厦高铁等重大项目前期工作。

　　2. 促进项目提速增效。完善领导挂钩、定期会商、问题专报、跟踪督办等工作机制，以“一月一活动”为抓手，强化正向激励，突出重点攻坚，推进一线考察干部，形成全市上下齐心协力、大抓项目抓大项目的浓厚氛围。优化投资计划管理，强化用地、用林、用工、资金等保障，集中优势资源推进重点项目建设。对重特大项目，成立工作专班，明确节点目标，“一对一”跟踪推进、全力突破。加强工程招投标、政府采购管理，进一步提高审批效率，加快项目落地建设。充分发挥苏区资源优势，高质量做好项目策划储备和申报工作，争取更多重大项目列入国家、省相关规划计划。

　　3. 实施精准招商。创新招商思路，找准优势互补、互利共赢的项目合作点，通过产业+资源、+技术、+人才、+市场等方式有效招商引资。创新招商方式，发挥招商公司作用，推行产业链招商、以商招商、要素招商、国企招商、军民融合招商、乡贤招商，引进实施一批大项目好项目。强化招商责任，坚持“一把手”带头招商、管行业必须管招商，将招商工作与绩效相挂钩，大力激活县（市、区）战斗单元作用，形成真招商招真商的工作氛围。坚持权责对等、优惠与产出挂钩，实行奖励政策与税收、就业贡献同步，提高招商实效。更加重视支持老企业增资扩产上新项目，要像招商引资一样予以优惠。更加重视服务业招商，像抓工业项目一样抓服务业项目，着力在文旅康养领域引进实施一批优质项目。

　　4. 依法推进征迁攻坚。依法征迁是加快建设、改善民生的必然需要。按照“依法依规、集中推进、先难后易、节点突破”要求，坚持宣传发动、思想工作、补偿安置、司法推动“齐头并进”，落实属地责任，强化街镇、部门协调联动，保障群众合法权益，抓好华龙社区改造、大洋片区综合改造、龙津湖安置地、龙岩高速东环线等重点项目征迁。创新供地方式，强化重点产业发展用地保障，集约节约用地，清理盘活闲置、低效土地，探索先租后让、租让结合供地，加快招商项目落地速度。

　　（三）着力民生民忧，提升群众获得感

　　民生无小事，枝叶总关情。增进民生福祉是发展的根本目的。必须突出补短板、促均衡、兜底线，认真办好一批为民办实事项目，切实解决好事关百姓切身利益的“头等大事”和影响百姓日常生活的“关键小事”。

　　1. 加快补齐教育短板。全面落实加强基础教育工作十条措施。加快中心城区20个应急性基础教育项目建设，确保龙岩一中锦山学校、市教科院附属小学、龙岩实验学校等8所学校秋季招生。深入推进“名校带动工程”和教育信息化建设，推行教师编制市域统筹试点，加强教师队伍建设，落实优教优酬、多教多酬，改善教师待遇，提高教育教学质量。继续推行阳光招生，实行公办义务教育积分制入学。新改扩建公办幼儿园20所，加大普惠性民办幼儿园认定及政府购买服务力度，鼓励社会资本开办普惠性学前教育。支持龙岩学院创建省示范性应用型本科高校。建立闽西职业技术学院产教融合长效机制。推进龙岩技师学院第二校区建设。

　　2. 提升医疗服务水平。实施提升医疗服务行动，建立完善检验结果互认、检查全预约等机制，优化看病结算流程，解决就诊、缴费排长队和看病时间短、停车难、乘电梯难等问题，切实改善百姓就医环境。加强医院护工的规范管理。扩大儿科、妇产科、老年科、精神卫生服务供给，实现每千名儿童儿科床位2.9张，每千分娩量产科床位21张。市第一医院分院、市中医院医技综合大楼等项目建成使用，规划建设龙岩人民医院东肖分院等项目。深化“三医联动”改革，持续推进医疗“创双高”，加强基层卫生人才队伍建设。完善全面二孩政策配套措施。鼓励社会资本办医，创办中高端医养结合机构。

　　3. 加大社会保障力度。完善就业创业服务体系，建设综合性人力资源超市，加强就业与用工信息互通，确保零就业家庭动态清零，全年新增城镇就业1.8万人以上。实现工伤保险参保全覆盖。医保大病专项救治病种扩大到21个以上。推进第三代社保卡就医、乘车、异地用卡等“一卡通办”便捷服务。鼓励中低价位、中小户型商品房开发供应，满足不同家庭住房需求。棚户区改造新开工5756套、基本建成6000套。深入推进全国居家和社区养老服务改革试点，总结推广乡镇敬老院连锁化运营经验，确保生活不能自理的特困人员集中供养率达40%以上，每千名老人拥有床位数35张。推行“互联网＋社会救助”模式，提高低保覆盖面，建立城市困难群体基本生活保障机制，切实做好托底工作。

　　4. 推动社会治理创新。持续推进更高水平平安龙岩建设，维护社会安定稳定。强化社会治安综合治理，打防并举、标本兼治，有效预防和治理违法犯罪，严防发生恶性群体性事件和个人极端刑事犯罪。学习运用“枫桥经验”，加强基层党组织、城乡社区群众自治组织建设，深入开展社会矛盾纠纷排查化解，有效管控化解重大矛盾隐患。提高政治站位，加大依法严惩、深挖彻查、综合治理、组织建设等工作力度，纵深推进扫黑除恶专项斗争。深入开展涉麻制毒、电信诈骗、农村赌博等突出问题整治。加强应急处突能力建设，推进公共安全视频监控“雪亮工程”。严格落实安全生产责任制，加强双重预防机制建设，夯实安全生产基层基础，坚决遏制重特大事故发生。深化“餐桌污染”治理，强化食品药品安全监管，创建国家食品安全示范市。完善应急管理机制，提高防灾减灾救灾能力。加大力度保障农民工工资支付。健全信用承诺、红黑名单、联合奖惩等诚信制度，推进公共信用平台建设。坚守底线，严格地方政府债务限额管理，有效防范和化解金融风险。

　　实施文化惠民工程，培育新型文化业态。加强优秀传统文化、非遗传承活用及重要文化遗迹遗存保护。推动全民健身和竞技体育协调发展。保障妇女、青少年、老年人、残疾人合法权益，关爱农村留守儿童健康成长。深化双拥共建，创建全国双拥模范城（县），关爱退役军人，进一步加强国防动员、后备力量和人民防空建设。高质量完成第四次全国经济普查工作。

　　（四）着力城乡协同，建设宜居美丽龙岩

　　城乡融合是提高发展平衡性和协调性的必然要求。按照“一市两区三组团”发展格局，强化以城带乡、城乡统筹，持续推进基础设施一体化和公共服务均等化。

　　1．拓展城市发展空间。实施市区两级联动培育新增长点行动，支持新罗区打造新增长区域，推进雁石、白沙产业园建设。支持永定区加快融入中心城市发展，建设坎市产业园，壮大产业支撑；推进靖永高速、双永高速坎市互通、快速通道延伸段、龙岩机场连接线等基础设施项目；开展结对帮扶，提升教育、医疗等公共服务水平。调整优化经开区经济管理、财税运行、收益分成等机制，赋予经开区更多审批权限，鼓励经开区与县（市、区）跨区域合作，做大做强特色优势产业。进一步创新厦龙合作区运营管理机制，促进产业项目加快落地。统筹抓好龙雁、高坎、古蛟三大组团开发建设，加快产业集聚，推进产城融合发展。大力激活壮大县域经济，支持上杭江滨宜居城市、武平山水园林精品城市、长汀历史文化名城、连城山水旅游度假城市、漳平“一江两岸”精美城市建设，形成各地奋勇争先、加快发展的局面。

　　2. 完善提升城市功能。以创建第六届全国文明城市为抓手，宣传实施文明行为促进条例，推进共建共治共享，提升城市文明水平。加快新一轮城市总规修编，完善中心城市现有规划“一张图”，强化“多规合一”成果运用。继续实施中心城市交通畅通工程，推进龙岩大道高架桥、龙岩高速东环线、金鸡路二期等项目建设，抓好罗桥、小溪桥、罗龙路高架、龙岩大道中心商圈连廊四大节点和天宇路口等6个拥堵点改造项目。继续实施中心城市绿化美化提升工程，重点推进龙岩洞公园和重要节点绿化改造提升，启动双洋路、莲花桥至华莲桥等绿道建设。加强城市供水保障，整合提升中心城区供（制）水企业，加快供水设施及水源点规划建设，新建改造供水管网100公里。实施防洪防涝和污水垃圾治理工程，建设污水管网120公里、雨水管网150公里。推广生活垃圾分类，加强餐厨垃圾管理。新改建城市公厕70座。加快中心城市建筑垃圾消纳场建设，鼓励建筑垃圾资源综合利用。提高城市精细化管理水平，完善智慧城管、智慧交通系统，严格落实“街长”“巷长”制，进一步加强和规范物业管理。综合运用行政、市场等手段，促进房地产市场平稳健康发展。

　　3．深入推进乡村振兴。继续打好精准脱贫攻坚战，推广激励性扶贫模式，提高医疗保障待遇，重点解决因病、因意外事故返贫问题，健全长效脱贫机制，确保现行标准下贫困人口稳定脱贫，扶贫开发工作重点县、重点乡镇和贫困村全部摘帽。研究解决收入水平略高于建档立卡贫困户的群体缺乏政策支持等新问题。强化乡村发展资金保障，推进涉农资金整合优化，实行“大专项+任务清单”管理模式。培育农业新型经营主体，培训新型职业农民，完善高校毕业生返乡就业创业、科技特派员等制度，促进各类资源要素向乡村流动。加快建设123个“千村整治、百村示范”美丽乡村和21个乡村旅游示范村。提升农村基础设施，加快“四好农村路”及农田节水灌溉、农村饮水安全、农村电网改造升级等项目建设，改造农村路网200公里。实施农村人居环境整治三年行动计划，全面建成乡镇生活污水处理设施并有效投入使用，完成35个以上行政村污水垃圾治理试点，完成70万平方米以上农房整治，县（市、区）各抓好5个以上试点示范村，努力破解乡村建设“有新房无新村”难题。深化农村集体产权制度改革，完成农村集体经济组织成员身份确认，试点县乡完成股份合作制改革，推进农村产权抵押贷款业务。加大力度支持革命基点村、少数民族乡村建设与发展。

　　4. 加强环境保护治理。持续抓好中央、省环保督察反馈问题整改。落实“三线一单”机制，建立生态环境管控体系。打好“碧水、蓝天、净土”保卫战，深入推进河（湖）长制，全面完成第二次全国污染源普查，加强重点排污源监控，突出抓好水污染防治、水源地保护、臭氧和PM2.5协同控制、土壤污染治理修复、废弃矿山生态恢复治理等工作，重点流域水质达到或优于三类比例95.5%以上，小流域水质达到或优于三类比例总体高于90%，境内无劣五类水质小流域，城市空气质量保持全省前列。

　　5．打响生态建设品牌。深化生态环境领域改革，落实领导干部自然资源资产离任审计、生态环境损害赔偿、生态系统价值核算等制度，全面完成国家生态文明试验区年度改革任务。提升拓展水土流失治理“长汀经验”和林改“武平经验”，推进龙岩国家现代林业科技示范园区和武平国家集体林业综合改革试验示范区建设，规划建设古田绿色小镇，打造一批绿水青山就是金山银山示范点，探索更多具有龙岩特色的生态文明建设新模式。

　　（五）着力改革创新，优化发展环境

　　深化改革、创新引领是高质量发展的根本动力。必须以更高标准、更实举措，推动相关改革走深走实，充分激发市场主体活力和社会创造力。

　　1. 推进审批服务便民化。坚持“对自己狠心、让百姓舒心”，优化提升e龙岩功能，推动政务服务流程优化再造，e龙岩手机端可办事项达400项以上，市、县两级各50个高频事项“一趟不用跑”、各100个高频事项“最多跑一趟”、审批服务事项网上可办率达90%以上。完善“马上就办”服务标准，分类设置服务窗口，设立惠企政策服务专区，70%以上审批服务事项实现“一窗”分类受理，群众凭身份证、企业凭营业执照即可办理便民服务事项。深化工程建设项目审批改革，完善并联审批机制，推行“一口受理”，推进多规合一、多测合一、多评合一、多审合一和区域评估。建立完善市本级重大项目效益评估制度，提高投资项目质量。

　　2. 激发创新创造活力。创新激励机制，加大创新投入，实施科技型中小企业培育工程，新增国家级高新技术企业25家、省级高新技术企业30家、省级科技小巨人领军企业30家。实施智能制造科技专项行动，支持企业加快信息化和工业化深度融合，推动200家工业企业“上云上平台”。鼓励企业发展主辅分离、个性定制等新模式，新增省级智能制造试点示范企业1家以上、服务型制造示范企业3家以上。深化与清华大学、厦门大学等知名高校及科研院所合作，发挥好厦门大学龙岩产教融合研究院等创新平台作用，新增5家省级以上研发机构。创新引才引智模式，完善人才使用和激励机制，支持职业院校与企业深化合作，培育和引进我市重点产业紧缺人才。弘扬工匠精神，评选第二届“龙岩工匠”。

　　3. 深化机构改革和国企改革。全面完成市县乡三级机构改革。赋予县、乡两级更多自主权，灵活有效调配行政资源。深入推进市属国企集团内部权属企业整合改革，撤并重组同质同类企业，二三级企业减少10%以上，促进资源、人才、技术等要素向企业主业集中、向优势企业集中。完善现代企业制度，增强投融资能力，强化风险管控，提高国资国企运行质量效益，在产业发展、项目建设中发挥“当先锋、打头阵”作用。

　　4. 提升对外开放水平。主动对接“一带一路”和海丝核心区建设，支持紫金矿业等企业“走出去”投资兴业、拓展市场。复制推广自贸区改革创新成果，创新监管和服务机制，营造更便利、更高效的投资环境。扩大龙台龙港龙澳合作和国际友城交流，在经贸、旅游、人文、教育、科技等方面深化对接合作。提升漳平台湾农民创业园和永定、武平海峡两岸交流基地建设水平。

　　三、建设人民满意的服务型政府

　　新时代、新任务对政府工作提出新的更高要求。我们将始终在习近平新时代中国特色社会主义思想指引下，牢记习近平总书记谆谆嘱托，按照全面从严治党要求，坚持党对一切工作的领导，坚持以人民为中心，坚持高质量发展落实赶超，担当负责、埋头苦干，把好思路做成好项目，以好项目支撑大发展。

　　心中有党，把政治建设摆在首位。不忘初心、牢记使命，传承红色基因，树牢“四个意识”，坚定“四个自信”，坚决做到“两个维护”，严守政治纪律和政治规矩。把讲政治落实到具体行动，始终做到党委有号召、政府有行动，党委有部署、政府抓落实。牢记业务工作有政治，加强对业务工作的政治把握，立足岗位，忠诚奉献，把实绩写在大地上，把项目落在坐标上。

　　心中有民，以群众评价衡量工作效能。发扬苏区干部好作风，深怀为民之心、多谋利民之策，持之以恒为民办实事办好事，尽心尽力解决事关群众切身利益的教育、医疗、交通、环保、治安等问题，持续增强百姓获得感。善于运用互联网技术和信息化手段开展工作，通过e龙岩推进“马上办、掌上办”，努力让群众、企业办事像网购一样方便。动员群众积极参与公共事务管理，加强对政府工作的监督。

　　心中有责，担当作为攻坚克难。面对矛盾不回避、遇到困难不退缩，多为闽西发展担当尽责、冲锋在前，对决定的工作部署，一抓到底、务求实效。深入开展机关作风整治，大力弘扬“马上就办、真抓实干”，以“滚石上山”的干劲攻坚克难。坚持问题导向和目标导向相统一，既鼓励大胆试、大胆闯，又坚持实事求是、善作善成。为担当者加油，为负责者鼓劲，常态化推动正向激励与逆向惩戒，让干好干坏不一样、干多干少不一样。改进督查考核，坚决纠正“过度留痕”现象，让基层有更多时间抓工作落实。完善政务清单管理制度，加强诚信政府建设，从严整治破坏发展环境的行为，打造一流营商环境。

　　心中有戒，立规严纪廉洁从政。严格遵守党的“六大纪律”，严格落实中央八项规定及实施细则精神，坚决防止和克服形式主义、官僚主义，坚决反对“表态多调门高、行动少落实差”。巩固拓展巡视整改成果，加强巡视巡察结果运用。自觉接受市人大及其常委会的监督和市政协的民主监督，认真执行人大决议决定，认真办好人大代表建议和政协委员提案并纳入政府决策。开展重点领域政府立法，严格公正文明规范执法，加强行政执法与刑事司法相衔接。加强法治教育和廉政教育，坚持用制度管权管事管人，防范工程建设、产权交易等重点领域廉政风险。加强审计监督，全面实施预算绩效管理，提高财政资金使用效益。厉行勤俭节约，严控“三公”经费和一般性支出，以政府过“紧日子”让群众、企业过“好日子”。

　　各位代表，伟大梦想不是等得来、喊得来的，而是拼出来、干出来的。让我们更加紧密团结在以习近平同志为核心的党中央周围，在省委、省政府和市委的坚强领导下，展现新担当，谋划新作为，锐意进取、苦干实干，奋力推进闽西各项事业迈上新台阶，以优异成绩迎接新中国成立70周年！
